# Comparison of plasma ctDNA and tissue/cytology-based techniques for the detection of *EGFR* mutation status in advanced NSCLC: Spanish data subset from ASSESS

**DOI:** 10.1007/s12094-018-1855-y

**Published:** 2018-04-05

**Authors:** E. Arriola, A. Paredes-Lario, R. García-Gomez, P. Diz-Tain, M. Constenla, C. García-Girón, G. Márquez, M. Reck, G. López-Vivanco

**Affiliations:** 1 Medical Oncology Department, Hospital del Mar, Passeig Marítim 25-29, 08003 Barcelona, Spain; 2 Medical Oncology, Hospital Universitario Donostia, San Sebastián, Spain; 3 Medical Oncology, Hospital General Universitario Gregorio Marañón, Madrid, Spain; 4 Medical Oncology, Complejo Asistencial Universitario de León, León, Spain; 5 Medical Oncology, Complexo Hospitalario de Pontevedra, Pontevedra, Spain; 6 Medical Oncology, Hospital Universitario de Burgos, Burgos, Spain; 7 Medical Affairs, AstraZeneca, Madrid, Spain; 8 Department of Thoracic Oncology Airway Research Center North (ARCN), German Centre for Lung Research (DZL), Grosshansdorf, Germany; 9 Medical Oncology, Hospital Universitario Cruces, Baracaldo, Spain

**Keywords:** Non-small-cell lung cancer, *EGFR* mutation, ctDNA, Plasma, Real-world, Liquid biopsy

## Abstract

**Purpose:**

The analysis of epidermal growth factor receptor (*EGFR*) mutations in many patients with advanced non-small-cell lung cancer (aNSCLC) has provided the opportunity for successful treatment with specific, targeted EGFR tyrosine kinase inhibitors. However, this therapeutic decision may be challenging when insufficient tumor tissue is available for *EGFR* mutation testing. Therefore, blood surrogate samples for *EGFR* mutation analysis have been suggested.

**Methods:**

Data were collected from the Spanish cohort of patients in the large, non-interventional, diagnostic ASSESS study (NCT01785888) evaluating the utility of circulating free tumor-derived DNA from plasma for *EGFR* mutation testing. The incidence of *EGFR* mutation in Spain and the level of concordance between matched tissue/cytology and plasma samples were evaluated.

**Results:**

In a cohort of 154 eligible patients, *EGFR* mutations were identified in 15.1 and 11.0% of tumor and plasma samples, respectively. The most commonly used *EGFR* mutation testing method for the tumor tissue samples was the QIAGEN Therascreen^®^ EGFR RGQ PCR kit (52.1%). Fragment Length Analysis + PNA LNA Clamp was used for the plasma samples. The concordance rate for *EGFR* mutation status between the tissue/cytology and plasma samples was 88.8%; the sensitivity was 45.5%, and the specificity was 96.7%.

**Conclusions:**

The high concordance between the different DNA sources for *EGFR* mutation testing supports the use of plasma samples when tumor tissue is unavailable.

**Electronic supplementary material:**

The online version of this article (10.1007/s12094-018-1855-y) contains supplementary material, which is available to authorized users.

## Introduction

Non-small cell lung cancer (NSCLC) represents 80–85% of all lung cancers and is a leading cause of cancer-related death worldwide [1]. It is often diagnosed at advanced stages (aNSCLC). However, the management of aNSCLC, which has been traditionally treated with systemic chemotherapy, has evolved in recent years with developments in genetic profiling and the identification of driver mutations. Investigations of the activating mutations of the epidermal growth factor receptor (*EGFR*) gene have resulted in specific therapies targeting these molecular abnormalities [2]. *EGFR* mutations are present in approximately 13% of European and 47% of Japanese patients [3]. In Spanish patients, *EGFR* mutations have been reported in 10–16% [4] of patients in some series and in 11.6% in a specific trial [5]. The first-generation reversible EGFR tyrosine kinase inhibitors (TKIs) gefitinib and erlotinib and the second-generation irreversible TKI afatinib elicit dramatic responses as a first-line treatment in *EGFR*-mutant aNSCLC patients [6–9]. Furthermore, EGFR TKIs have demonstrated superior efficacy in conjunction with chemotherapy in patients with *EGFR* mutation-positive aNSCLC [10–14]. However, patients will only benefit from these agents if procedures to perform *EGFR* mutation testing are effectively implemented in a clinical setting.

A variety of methodologies are available for *EGFR* mutation testing [15, 16], and direct DNA sequencing is considered the “gold standard” [17]. However, recently developed commercial real-time quantitative PCR kits have successfully increased sensitivity, reducing the amount of tumor DNA required to detect the mutation in a patient sample [15]. These molecular techniques are subject to some limitations. The primary obstacle is a lack of sufficient tumor tissue sample. Other factors include the diverse accuracy of testing techniques [18] and, logistically, the limited number of testing laboratories and poor reimbursement by healthcare systems [19]. Cytology samples have proven to be a valid source of material for mutation testing [18, 20, 21]. Recently, the assessment of *EGFR* mutations in surrogate samples, such as serum or plasma, has gained importance [22]. In the ASSESS study [23], we observed high concordance of mutation status between matched biopsy/cytology and plasma samples, suggesting that circulating free tumor-derived DNA (ctDNA) can be isolated from the plasma or serum and could serve as a feasible sample for real-world *EGFR* mutation analysis. These findings were linked to robust and sensitive DNA extraction and mutation analysis methodologies. ctDNA comprises < 1% of total circulating free DNA [24]; when the previous analysis included only the subset of patients for whom DNA was re-extracted with an optimized method, the overall concordance rate and sensitivity further increased.

The present analysis from ASSESS, which originally included European and Japanese patients, aims to increase the understanding of local practices for *EGFR* mutation testing and the incidence of *EGFR* mutations in a cohort of the Spanish population under a real-world diagnostic framework.

## Methods

ASSESS was a large, multicenter, non-interventional, diagnostic study (ClinicalTrials.gov NCT0178588) designed to evaluate different sample types as ctDNA sources as well as to perform *EGFR* mutation testing in patients with aNSCLC in a real-world diagnostic setting. The study was conducted in 8 centers in Japan and 48 centers in 7 European countries, including Spain. The study protocol was approved at all participating sites. The study was conducted according to the principles of the Declaration of Helsinki and the ICH Guidelines for Good Clinical Practice and was based on the applicable regulatory requirements for non-interventional studies and AstraZeneca’s policy on bioethics and human biological samples. All patients provided written informed consent. The study design has been described in full elsewhere [23] and is summarized here.

### Study population

Eligible patients in European countries met the following inclusion criteria: age ≥ 18 years; either histologically or cytologically confirmed local advanced NSCLC (stage IIIA/B [American Joint Committee on Cancer staging system]); treatment naïve and unsuitable for either curative treatment or chemoradiotherapy. Individuals with metastatic disease (stage IV) and patients with recurrent disease after previous curative treatment (resection and/or adjuvant chemotherapy) were included. DNA extraction from a tumor sample and from a fresh blood (plasma) sample for *EGFR* mutation testing was mandatory upon enrollment. The blood sample was collected prior to the initiation of any treatment.

### Study procedures

The *EGFR* mutation testing methodology has been previously published [23]. In brief, centers conducted *EGFR* mutation testing with diagnostic tumor samples according to their local practices. All samples underwent histopathological review to ensure that they were adequate for use and were classified according to World Health Organization (WHO) guidelines [25]. In Spain, DNA extraction from blood samples and subsequent mutation testing were performed in one designated laboratory using Fragment Length Analysis + PNA Clamp technology.

The primary objective of the study was to determine the level of concordance between the *EGFR* mutation status obtained from tumor samples and that obtained from blood samples. The concordance rate, sensitivity, specificity, positive predictive value (PPV), and negative predictive value (NPV) were calculated for the tissue/cytology and plasma samples, and the exact two-sided 95% confidence interval (CI) was obtained. The false-positive rate was defined as an *EGFR* mutation-positive result in plasma but mutation-negative result in tissue/cytology. The opposite definition was used for the false-negative rate. Secondary variables aimed to describe the mutation incidence and subtypes in Spain, correlations between *EGFR* mutation status and demographic data or disease status, and the treatment decisions following *EGFR* mutation testing.

### Statistical analyses

The sample size calculation for this study is reported elsewhere [23]. In the analysis presented herein, only the subset of patients recruited in Spanish centers was included. *EGFR* mutation testing practices, *EGFR* mutation incidence and subtypes, and information regarding treatment choices were summarized using appropriate descriptive statistics. For the concordance rate between the EGFR mutation status in the tumor and plasma samples, we calculated the sensitivity, specificity, and the positive and negative predictive values.

## Results

### Patients

The ASSESS study included a total of 1311 (1288 eligible) patients, 158 (154 eligible) of whom were recruited in Spain. Four patients were excluded in this subanalysis because they did not fulfill the inclusion/exclusion criteria. The patient demographics and information regarding their NSCLC disease status are listed in Table 1; the majority were men (74.7%), with a mean age of 64.0 ± 10.13 years, and 72.7% were diagnosed with adenocarcinoma.

**Table 1:** Patient demographics and disease information of the total and tumor-evaluable Spanish populations

	Evaluable population	Tumor-evaluable population	
		Mutated	Wild-type
Age, mean ± SD	64.0 ± 10.13	69.9 ± 11.81	62.7 ± 9.58
Sex (male), *n*/*N* (%)	115/154 (74.7)	11/22 (50.0)	93/121 (76.9)
Race, *n*/*N* (%)			
Caucasian	154/154 (100)	22/22 (100)	121/121 (100)
Asian	0/154 (0.0)	0/22 (0.0)	0/121 (0.0)
Smoking status			
Non-smoker^a^, *n*/*N* (%)	27/154 (17.5)	15 (68.2)	12 (9.9)
Current smoker, *n*/*N* (%)	53/154 (54.4)	2 (9.1)	49 (40.5)
Pack-years^b^, mean ± SD	168.4 ± 222.32	61.2 ± 60.99	170.8 ± 221.67
Time since first diagnosis (months), mean ± SD	4.2 ± 17.61	2.4 ± 6.12	4.8 ± 19.67
Treatment naïve, *n*/*N* (%)	101/154 (65.6)	14/22 (63.6)	78/121 (64.5)
Histology, *n*/*N* (%)			
ADC	112/154 (72.7)	20/22 (90.9)	88/121 (72.7)
Non-ADC	40/154 (26.0)	2/22 (9.1)	32/121 (26.4)
WHO performance status, *n*/*N* (%)			
0	38/154 (24.7)	7/22 (31.8)	28/121 (23.1)
1	88/154 (57.1)	10/22 (45.5)	71/121 (58.7)
2	25/154 (16.2)	4/22 (18.2)	20/121 (16.5)
3	3/154 (1.9)	1/22 (4.5)	2/121 (1.7)
4	0/154 (0.0)	0/22 (0.0)	0/121 (0.0)
TNM (AJCC) stage status, *n*/*N* (%)			
IIIA	9/154 (5.8)	3/22 (13.6)	4/121 (3.3)
IIIB	9/154 (5.8)	0/22 (0.0)	7/121 (5.8)
IV	136/154 (88.3)	19/22 (86.4)	110/121 (90.9)
TNM (AJCC) stage IV, *n*/*N* (%)			
M1a	20/154 (13.0)	5/22 (22.7)	15/121 (12.4)
M1b	51/154 (33.1)	6/22 (27.3)	41/121 (33.9)

*ADC* adenocarcinoma, *AJCC* American Joint Committee on Cancer, *SD* standard deviation, *TNM* tumor-node-metastasis, *WHO* World Health Organization

^a^Non-smoker: less than 100 cigarettes in his or her lifetime

^b^Pack-years: (number of cigarettes smoked per day × number of years smoked)/20

### Tumor sampling and *EGFR* mutation testing procedures

The biopsy samples were most commonly obtained when the patient received the current diagnosis (109/146 [74.7%]) and from the primary tumor (109/146 [74.7%]). The majority were collected from the lung (101/146 [69.2%]); smaller percentages were samples collected from the lymph nodes (12/146 [8.2%]) and pleura (9/146 [6.2%]). The most common method for sample collection was bronchoscopy (50/146 [34.2%]) followed by core-biopsy (30/146 [20.5%]); a cytology sample was obtained for analysis in 27/146 (18.5%).

Tumor tissue and cytology samples were either unavailable or insufficient for 11 (7.7%) patients. Information related to the procedures for *EGFR* mutation testing is shown in Table 2. The most commonly used *EGFR* mutation testing method for the tumor tissue samples was the QIAGEN therascreen^®^ EGFR RGQ PCR kit (QIAGEN, Manchester, UK; *n* = 76; 52.1%) followed by the Roche cobas^®^ EGFR Mutation Test (Roche Molecular Diagnostics, Pleasanton, CA; *n* = 41; 28.1%). The *EGFR* mutation testing method used for all plasma samples was Fragment Length Analysis + PNA LNA Clamp. The overall mean testing turnaround times for detection were 19.2 ± 43.69 days with a testing success rate of 97.9% for methodologies applied to tumor samples and 35.5 ± 20.33 days with a mutation testing success rate of 100% for plasma samples.

**Table 2:** Evaluation of EGFR mutation testing performed using tissue/cytology and plasma samples

	Tissue/cytology	Plasma
Samples available, *n*/*N* (%)	146/154 (94.8)	154/154 (100)
Testing not performed, reasons, *n*/*N* (%)		
Insufficient material	5/154 (3.2)	0/154 (0.0)
Test not ordered/requested	2/154 (1.3)	0/154 (0.0)
Other	1/154 (0.6)	0/154 (0.0)
*EGFR* mutation, *n*/*N* (%)		
Negative	121/146 (82.9)	137/154 (89.0)
Positive	22/146 (15.1)	17/154 (11.0)
Unknown	3/146 (2.1)	0/154 (0.0)
*EGFR* mutation subtype, *n*/*N* (%)		
Exon 19 deletions	8/22 (36.4)	6/17 (35.3)
L858R alone	10/22 (45.5)	4/17 (23.5)
Exon 19 + T790M	0/22 (0.0)	0/17 (0.0)
L858R + T790M	0/22 (0.0)	1/17 (5.9)
T790M alone	0/22 (0.0)	3/17 (17.6)
T790M + other^a^	1/22 (4.5)	1/17 (5.9)
Other	3/22 (13.6)	2/17 (11.8)

^a^Any other mutation that occurred in combination with T790M, excluding L858R or Exon 19 deletions

### *EGFR* mutation incidence and concordance rate between sampling types

Positive mutations of the *EGFR* gene were identified in 15.1 and 11.0% of the tumor and plasma samples, respectively (Table 2). When the data were categorized by tumor histology type, *EGFR* mutations were detected in 20/108 (18.5%) tumor samples and 14/112 (12.5%) plasma samples from adenocarcinoma patients and in 2/34 (5.9%) and 3/40 (7.5%) samples from non-adenocarcinoma individuals, respectively. The most common mutation subtypes were the single L858R substitution and exon 19 deletions in both tumor specimens (10/22 [45.5%] and 8/22 [36.4%], respectively) and plasma samples (4/17 [23.5%] and 6/17 [35.3%], respectively; Table 2).

The concordance rate between the *EGFR* mutation status in the tumor and plasma samples was 88.8% (95% CI 82.5–93.5). The calculated sensitivity was 45.5% (95% CI 24.4–67.8), with a specificity of 96.7% (95% CI 91.8–99.1), PPV of 71.4 (95% CI 41.9–91.6) and NPV of 90.7 (95% CI 84.3–95.1). False-positive results were defined as *EGFR* mutation-positive in plasma and mutation-negative in tumor samples and were observed in 4 patients (rate of 2.8%). Characteristics of these patients are listed in Supplementary Table S1. The corresponding false-negative rate was 8.4%, corresponding to 22 patients.

### Treatment strategy according to *EGFR* mutation status

The first-line treatment strategies according to *EGFR* mutation status for the population with known tumor mutation status are described in Table 3. Most patients who were *EGFR* mutation-positive received EGFR TKIs (19/22 [86.4%]), including gefitinib (42.1%), erlotinib (36.8%) and afatinib (21%). In those patients negative for an *EGFR* mutation, systemic chemotherapeutic strategies were preferred. The primary reason for treatment selection was *EGFR* mutation status in 79 patients (51.6%).

**Table 3:** First-line treatment strategy according to EGFR mutation testing results

	*EGFR* positive	*EGFR* negative	Total^a^
EGFR tyrosine kinase inhibitors, *n*/*N* (%)			
Gefitinib	8/22 (40.0)	0/121 (0.0)	8/143 (5.6)
Erlotinib	7/22 (35.0)	0/121 (0.0)	7/143 (4.9)
Afatinib	4/22 (20.0)	0/121 (0.0)	4/143 (2.8)
Systemic chemotherapy, *n*/*N* (%)			
Pemetrexed	1/22 (5.0)	60/121 (55.6)	61/143 (42.7)
Carboplatin	0/22 (0.0)	43/121 (39.8)	43/143 (30.1)
Cisplatin	1/22 (5.0)	39/121 (36.1)	40/143 (28.0)
Vinorelbine	1/22 (5.0)	39/121 (36.1)	40/143 (28.0)
Paclitaxel	0/22 (0.0)	5/121 (4.6)	5/143 (3.5)
Gemcitabine	0/22 (0.0)	3/121 (2.8)	3/143 (2.1)
Docetaxel	0/22 (0.0)	1/121 (0.9)	1/143 (0.7)
Monoclonal antibodies, *n*/*N* (%)			
Bevacizumab	0/22 (0.0)	3/121 (2.8)	3/143 (2.1)
Radiotherapy, *n*/*N* (%)	1/22 (4.5)	19/121 (15.7)	20/143 (14.0)
Investigational agent, *n*/*N* (%)	0/22 (0.0)	2/121 (1.9)	2/143 (1.4)

^a^Evaluable population with available plasma samples

## Discussion

Identification of mutations in aNSCLC is essential to guide oncologists in molecular-based treatment decisions. Several clinical trials have previously suggested that plasma ctDNA detection is an appropriate surrogate marker of *EGFR* mutation status [26–28] In a real-world setting, the diagnostic ASSESS study showed the utility of plasma-derived ctDNA samples and good concordance based on results for 1162 matched tissue/cytology samples (89%; sensitivity 46%, specificity 97%, PPV 78%, and NPV 90%)0 [23]. Despite this good concordance, the sensitivity in our analysis was low. Thus, the pooled sensitivity was higher in two recent meta-analyses of EGFR mutation status concordance between matched tissue/cytology and plasma samples (62–67%) [29, 30]. However, it should be taken into account that sensitivity may greatly differ between a real-world study, such as the ASSESS, and clinical trials, where testing is generally well controlled and performed in a single, central laboratory, and patients are more homogenous regarding their disease stage. The present post hoc analysis of the Spanish subset of patients in the ASSESS study further confirms the utility of ctDNA analysis in plasma for the evaluation of *EGFR* mutation status when tumor tissue is unavailable or exhausted.

However, *EGFR* mutation testing practices are subject to well-described challenges. The capacity to detect mutations by ctDNA appears to be limited in some patients. We detected *EGFR* mutations in 15.1% of the aNSCLC patients according to tumor samples and in 11.0% according to plasma ctDNA. A lower *EGFR* mutation detection rate by ctDNA has been described previously [23, 31]. By contrast, in four cases, the *EGFR* mutation status was positive in the plasma sample and negative in the tumor sample. It is unclear if this is due to a false-negative reading in the tumor sample or false-positive reading in the ctDNA sample. Usually, false negative is found in plasma sample because of sensitivity of method and limited copy number of ctDNA. However, the heterogeneity of the mutation in the tumor sample may not be captured by biopsy analysis but may be recognized in plasma ctDNA analysis. Interestingly, 2/4 tumor samples with these discordant results were cytology blocks that may not be representative of the whole tumor genetic events and may represent heterogeneity within the tumor tissue. Moreover, the amount of DNA available from these samples may have been insufficient for detection by the PCR-based technique. Notably, 2/4 tumors harbored T790M mutations that may not be present in all tumor cells and again heterogeneity in the tumor could explain these divergent results.

In our study, discrepancies in the identification of distinct mutation subtypes were observed depending on the sample type. T790M was detected in four patients by ctDNA analysis but in only 1 patient according to the tumor tissue results. By contrast, six L858R mutations were missed in the plasma assessment, consistent with previous reports of a lower detection rate of L858R [27, 28]. A recent meta-analysis showed a wide variation in T790M detection rates, which depended on how sensitive or feasible was the technique for detection [32]. Thus, these discrepancies might reflect differences in the sensitivities of the technologies for detecting each mutation type.

Globally, in these discordant cases, clinical judgment should prevail and there are now multiple experiences of therapeutic decisions based on plasma ctDNA results where patient outcomes are concordant with expected behavior from these tumors with targeted treatments. However, whether these discordances add information to our clinical practice remains undetermined.

The use of highly sensitive detection kits is essential to avoid false-negative and false-positive results. The mutation-detecting Sanger sequencing method has been established as the ‘gold standard’ for *EGFR* mutation testing [33], but the requirement of high-quality tumor samples and the longer turnaround time limit its utility [34, 35]. Amplification refractory mutation system (ARMS) based on PCR technology has been developed and is increasingly accepted in clinical practice. Alternatively, mutant-specific immunohistochemistry (IHC) or bead emulsion amplification and magnetics (BEAMing) has been proposed [31, 36]. Despite technical advances, variable concordance rates, sensitivity and specificity between blood-based and tumor samples for *EGFR* mutation detection have been reported, as shown in the global ASSESS study [23].

## Conclusion

In the subset of Spanish patients in the ASSESS study, the prevalence of the *EGFR* mutation rate and the demographic data was as expected for a Caucasian population. The good concordance (almost 90%) between the different DNA sources supports the use of plasma samples when tumor tissue is not available. However, this work highlights some limitations for *EGFR* mutation testing in the real-world setting and adds information on observed discordant results. In addition to the wide variety of testing techniques available and the differences in their sensitivity rates, this work emphasizes the importance of standardization and appropriately trained laboratories to reliably perform molecular characterization of aNSCLC patients.

## Electronic supplementary material

Below is the link to the electronic supplementary material.

Supplementary material 1 (XLS 20 kb)
